# Hyperlocal environmental data with a mobile platform in urban environments

**DOI:** 10.1038/s41597-023-02425-3

**Published:** 2023-08-05

**Authors:** An Wang, Simone Mora, Yuki Machida, Priyanka deSouza, Sanjana Paul, Oluwatobi Oyinlola, Fábio Duarte, Carlo Ratti

**Affiliations:** 1 Senseable City Lab, Department of Urban Studies and Planning, Massachusetts Institute of Technology, Cambridge, USA; 2 Department of Computer Science, Norwegian University of Science and Technology, Trondheim, Norway; 3 Department of Urban and Regional Planning, University of Colorado Denver, Denver, USA

**Keywords:** Environmental monitoring, Environmental impact, Developing world

## Abstract

Environmental data with a high spatio-temporal resolution is vital in informing actions toward tackling urban sustainability challenges. Yet, access to hyperlocal environmental data sources is limited due to the lack of monitoring infrastructure, consistent data quality, and data availability to the public. This paper reports environmental data (*PM*, *NO*_*2*_, temperature, and relative humidity) collected from 2020 to 2022 and calibrated in four deployments in three global cities. Each data collection campaign targeted a specific urban environmental problem related to air quality, such as tree diversity, community exposure disparities, and excess fossil fuel usage. Firstly, we introduce the mobile platform design and its deployment in Boston (US), NYC (US), and Beirut (Lebanon). Secondly, we present the data cleaning and validation process, for the air quality data. Lastly, we explain the data format and how hyperlocal environmental datasets can be used standalone and with other data to assist evidence-based decision-making. Our mobile environmental sensing datasets include cities of varying scales, aiming to address data scarcity in developing regions and support evidence-based environmental policymaking.

## Abstract (summary)

**Table:** 

Measurement(s)	*Particulate matter*, gaseous pollutants
Technology Type(s)	Low-cost environmental sensing platform (City Scanner)
Sample Characteristic - Environment	City
Sample Characteristic - Location	Global

## Background & Summary

Rapid urbanization has been posing new sustainability challenges to planners, engineers, scientists, and citizens in a climate change era. Hyperlocal environmental data are desirable for academics and practitioners to identify exposure hotspots, understand the spatial distribution of urban air pollution, and support evidence-based climate change mitigation. Yet, hyperlocal data acquisition remains a challenge in both developed and developing regions. Among all urban environmental data, air pollution data is one of the most challenging to monitor due to its high spatial and temporal variability. In cities, air pollution emission sources are diverse, and emission dispersion is highly volatile^1^. In recent years, mobile monitoring has been playing an increasingly important role in complementing traditional monitoring methods, such as stationary monitoring and satellite remote sensing^2–4^. It provides a highly scalable alternative to operate in various urban environments while generating high-resolution data.

Mobile air quality measurement techniques are documented in a rapidly expanding body of literature. The most notable ones include a series of studies conducted in collaboration with Google Street View cars in Houston, the San Francisco Bay Area, Amsterdam, Copenhagen, and London (https://www.google.com/earth/outreach/special-projects/air-quality). Reference and research-grade air monitors were carried around in cities, repetitively measuring most street segments in periods from months to years. Their raw data were partially published via a third-party online database and API (https://explore.openaq.org). While the spatial and temporal coverage was extensive, Google’s air monitoring campaigns were focused on populous urban areas with good street view image sampling density. Mobile monitoring instruments and laboratories operated by academics are another important air quality data source. Nonetheless, the final outputs for such deployment are often scientific papers and reports, commonly without publishing the full dataset^5–8^. Even though some papers have attached raw data files, there is less consistency in data quality from project to project, given the difference in study scope, instrumentation, personnel, sampling methods, and data validation. Another important air quality data pool is provided with the wider adoption of low-cost air sensing technology, citizen science, and crowd-sourced monitoring campaigns^9^. Moreover, lacking a unified, open-sourced channel to index and retrieve data from individual studies has created a substantial hurdle for non-academics to access and make further use of these data outside of scientific publications.

Our work targets mitigating the gap in the availability of hyperlocal environmental data in both developed and developing regions with three reproducible and low-cost advantages with consistent instrumentation, sampling methods, and data validation procedure^10^. We first introduce the mobile platform we used in real-world deployments in global cities and how its custom configurations can best serve these applications. We further present validated environmental sensing data, focusing on calibrated air monitoring data, from several mobile monitoring deployments in various urban contexts from the Middle East to North America in both developed and developing regions. Lastly, we demonstrate typical use cases of air pollution data by themselves and other data sources in New York City, including emission hotspots inference and air quality predictions. Our results agree well with the standing regulatory air quality prediction maps, proving the validity of our data collection and calibration methodology. This paper is timely for environmental practitioners and researchers to reflect on traditional air quality data collection approaches and publication, providing a good example for future practices.

## Methods

### Instrumentation

**Fig. 1:**
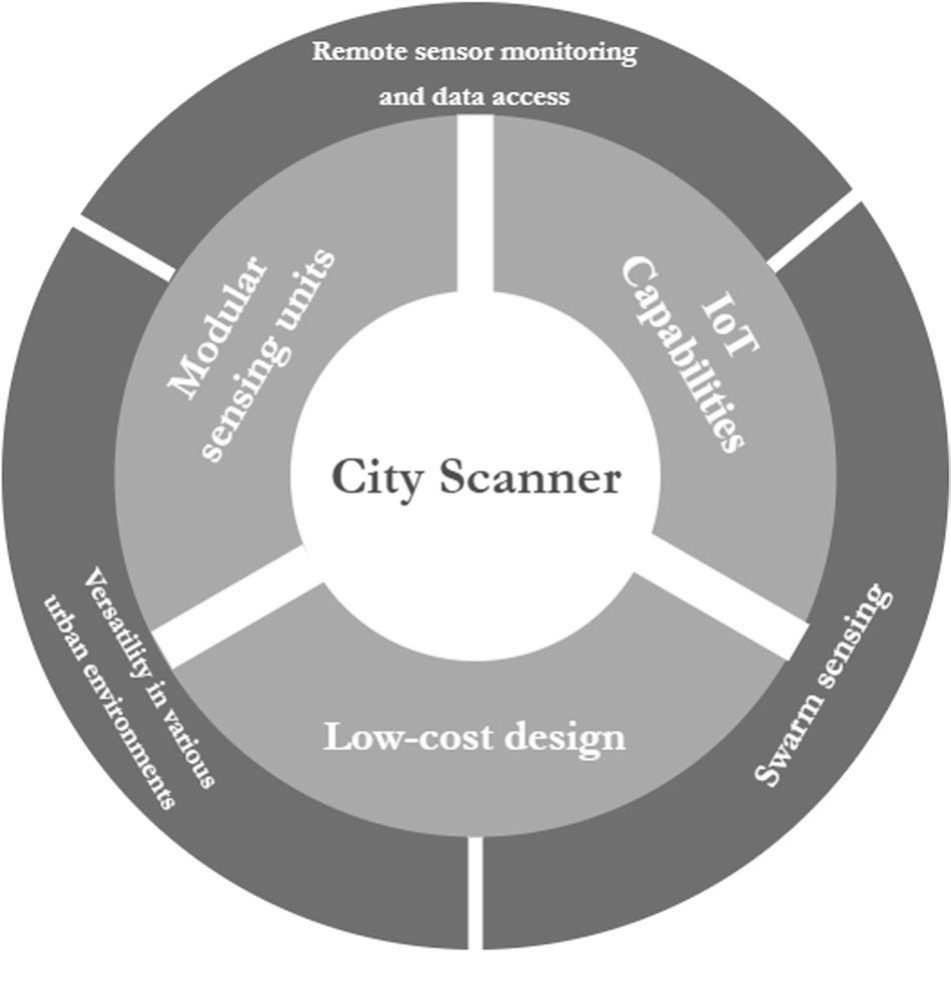
The City Scanner design boundary (inner) and function boundary (outer).

Air quality datasets in this study were collected using our self-designed and manufactured City Scanner (CS) mobile sensing platform^11,12^. It aims to enable large-scale environmental sensing tasks using existing urban fleets, such as taxis, buses, and municipal service vehicles, as sensing nodes. We present our sensor design, which is based on three concepts summarized in Fig. 1: low-cost design, modular sensing units, and Internet-of-Things (IoT) capabilities. Our low-cost design follows the definition of a low-cost air sensor by the US Environmental Protection Agency (EPA), which sets an upper-cost limit of $2,500 (https://www.epa.gov/air-sensor-toolbox/how-use-air-sensors-air-sensor-guidebook). CS’s modular sensing units allow users to easily customize their sensing capabilities or a range of environmental sensing applications. CS is IoT-enabled, where collected air quality data and device status can be instantly streamed to the cloud for storage and analysis via a cellular network. As shown in Fig. 1, the combinations of each two design concepts serve three major functions: a) monitoring individual sensor’s status and remote, instantaneous access to data, b) versatility in complex urban environments balancing device energy consumption, cost, and data requirement, and c) a vision for swarm sensing, where a CS fleet operates in coordination to reach real-time city scanning. Only a few sensors are required to sense a large urban space^13^.

**Fig. 2:**
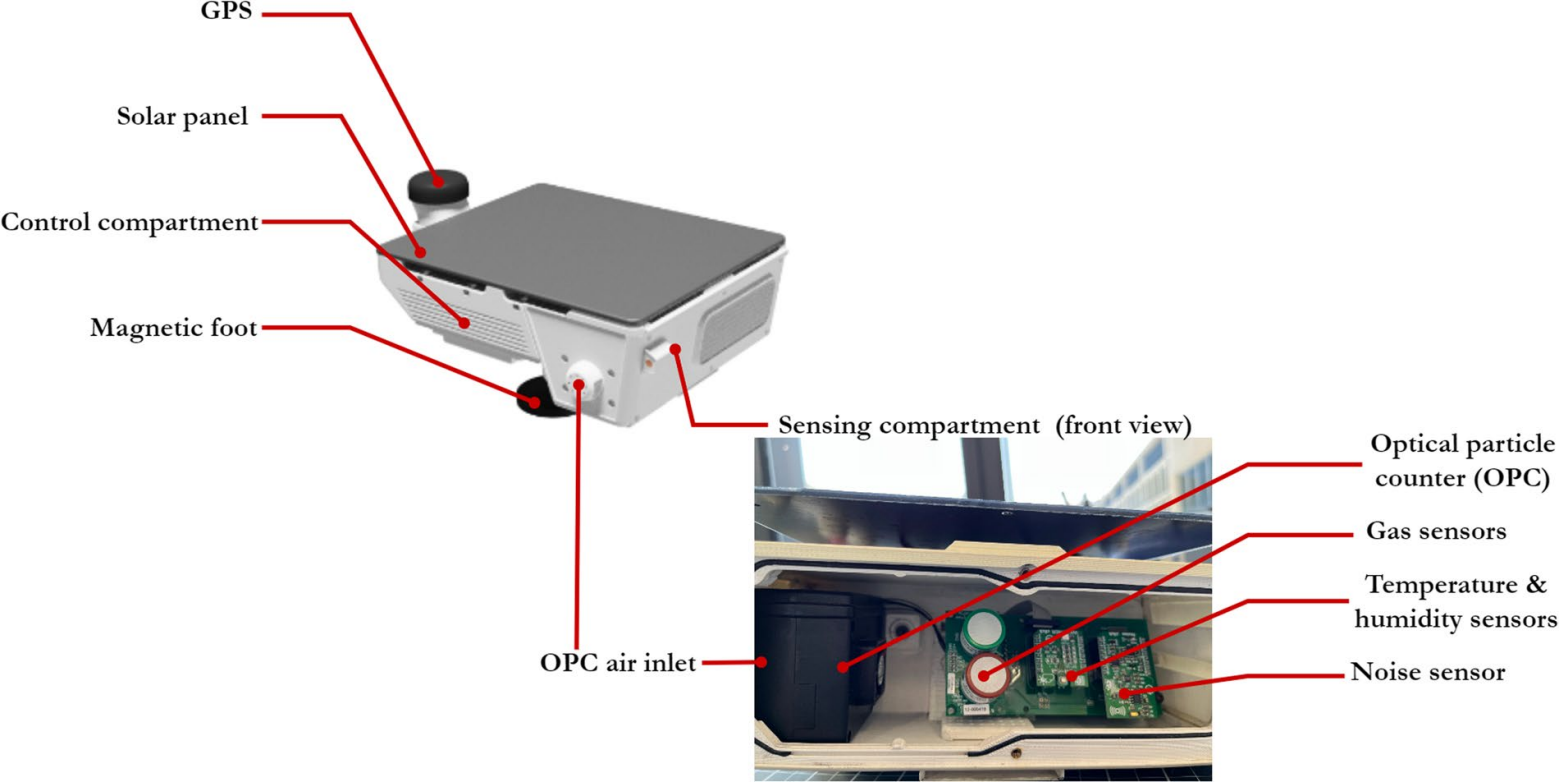
Whiteburn II basic configuration.

Specifically, each CS has two major compartments: the control and sensing compartments. The control compartment houses the motherboard, the data communication and local storage system, and the energy and thermal performance management system. The sensing compartment is more relevant to data collection and is detailed in this section. Figure 2 illustrates the basic configuration of the current CS iteration, named “Whiteburn II”, focusing on the sensing compartment in the front view.

In the standard configuration of the sensing compartment, a low-cost Alphasense OPC-N3 optical particle counter is adopted for *particulate matter* (*PM*) concentration measurement. It counts the number of particles by emitting a laser beam through the air flow being drawn in, so that by counting the pulses of light scattered by particles in the airflow, OPC can infer the number of particles of different sizes. This technique has been widely adopted in academic and citizen science projects^14,15^. Two gas sensors can be hosted in the sensing compartment simultaneously. In the sensor deployments reported in this study, we used Alphasense’s electrochemical gas sensors for *CO*, *NO*_*2*_, and *SO*_*2*_. The surface material on an electrochemical gas sensor reacts with the target gaseous pollutant, which results in an electric current that passes from the working to the reference electrode. The current is measured and is proportionate to the target pollutant’s concentration. CS has a basic platform with an OPC and the sensing capability of up to two gaseous pollutants. The platform is also equipped with basic environmental sensing capabilities, including ambient air temperature and relative humidity sensing. The weather sensors are mounted on the mikroBUS^TM^ socket, comprising a pair of 1 × 8 female headers. The pinout consists of three groups of communications pins (SPI, UART, and I2C), six additional pins (PWM, interrupt, analog input, reset, and chip select), and two power groups (+3.3 and 5 V). Table 1 documents a list of environmental sensors that are currently being used, which can be easily substituted as long as the data communication protocols conform.

**Table 1:** Environmental sensor specifications.

Sensor	Sensing subject	Unit cost	Substitution
Alphasense OPC-N3	*Particulate matter* count and estimated mass concentration	$350	With a thermal camera
Alphasense NO_2_-A4	Nitrogen dioxide	$50	With other gas sensors
Bosh BME-280	Temperature, humidity	$20	N/A

### Data collection campaigns

We used the CS platform version “Whiteburn II” for high spatio-temporal resolution data collection, which is designed as a plug-and-play environmental sensing platform. After a full charge, each CS was mounted on the roof of a vehicle, whose fleet information is presented in Table 2. The CS should be placed with the OPC air inlet facing sideways and the sensing compartment facing the direction of driving. This orientation minimizes the influence of vehicle speed on OPC air intake, while exposing the passive sampling gas sensors as much as possible to ambient air. We adopted an opportunistic data collection method, where CS data collection is not the main purpose of driving. It indicates that we do not have prescribed routes for the vehicles to follow. To avoid spatial and temporal biases of sampling (some places and some time slots are oversampled), each data collection campaign has been conducted in a sufficiently long period, lasting at least two months. The sampling period and area are also dependent on the study scope of each deployment. In all, our platform design and deployment protocols are simple to apply and easy to use, which intend to make environmental sensing available to a larger population and as many communities.

**Table 2:** Deployments in three cities.

City	Sensing target pollutants	Sensing fleet	Duration
New York City, US	*Particulate matter*, particle size distribution, *NO*_*2*_	Five municipal vehicles driven by park rangers	Jan. 2020 – Feb. 2020; Sep. 2021 – Dec. 2021
Boston, US	*Particulate matter*, particle size distribution, *NO*_*2*_	One mobile environmental laboratory	Feb. 2022 – Apr. 2022
Beirut, Lebanon	*Particulate matter*, particle size distribution	Two taxis	Feb. 2022 – Jun. 2022

Two deployments in New York City target the Bronx borough, with 2 million residents, mostly ethnic and racial minorities. The Bronx is disproportionately exposed to air quality hazards, as are many other vulnerable neighborhoods which are overburdened with environmental issues^16^. The region is covered by four reference stations operated by the New York State Department of Environmental Conservation, only two out of which measure gaseous pollutants in addition to *PM*. The New York City Department of Health designed New York City Community Air Survey (NYCCAS) with a finer air monitoring network of high-quality but not reference-grade sensors since 2008 (https://www.nyc.gov/site/doh/data/data-sets/air-quality-nyc-community-air-survey). NYCCAS runs fifteen monitoring sites in the Bronx, collecting hourly *PM*_*2.5*_, *black carbon*, *NO*, and *NO*_*2*_ concentrations once per season. Our Bronx deployments worked jointly with these local authorities to complement the existing network, providing more details on air quality’s spatial and temporal variability. Moreover, mobile air quality measurements are useful for quantifying air pollution exposure disparities at a high spatio-temporal resolution, which advises equitable and just air pollution mitigation plans.

In the Boston deployment, we collected hyperlocal air quality data in a neighborhood north of the Boston Logan International airport. We mounted CS units on a research-grade mobile environmental laboratory, which measures real-time *particulate matter* and *NO*_*x*_ concentrations, whose validity has been proved in previous publications^17,18^. We contrasted CS-collected data with high-quality mobile laboratory data, demonstrating the transferability and robustness of our mobile air quality sampling approach.

The final deployment presented in this study was conducted in Beirut, the capital of Lebanon. The country faces a severe economic collapse and has been suffering from air pollution from diesel generators, as their centralized power grid operates only a few hours per day. Currently, there is no government-regulated air quality monitoring infrastructure. To our knowledge, our collaborators at American University Beirut run the only research-grade air monitoring site, measuring *PM* and gaseous pollution. The extremely sparse air monitoring network cannot provide much useful information to tackle the deteriorating local air quality problem. In this case, our deployment aims to address the local air quality data gap in a data and resource-scarce urban environment. In all, our CS deployments proved the accuracy, validity, durability, and versatility in a variety of uncontrolled urban environments serving various purposes, including air quality management, climate change mitigation, citizen engagement, and knowledge dissemination.

## Technical Validation

**Fig. 3:**
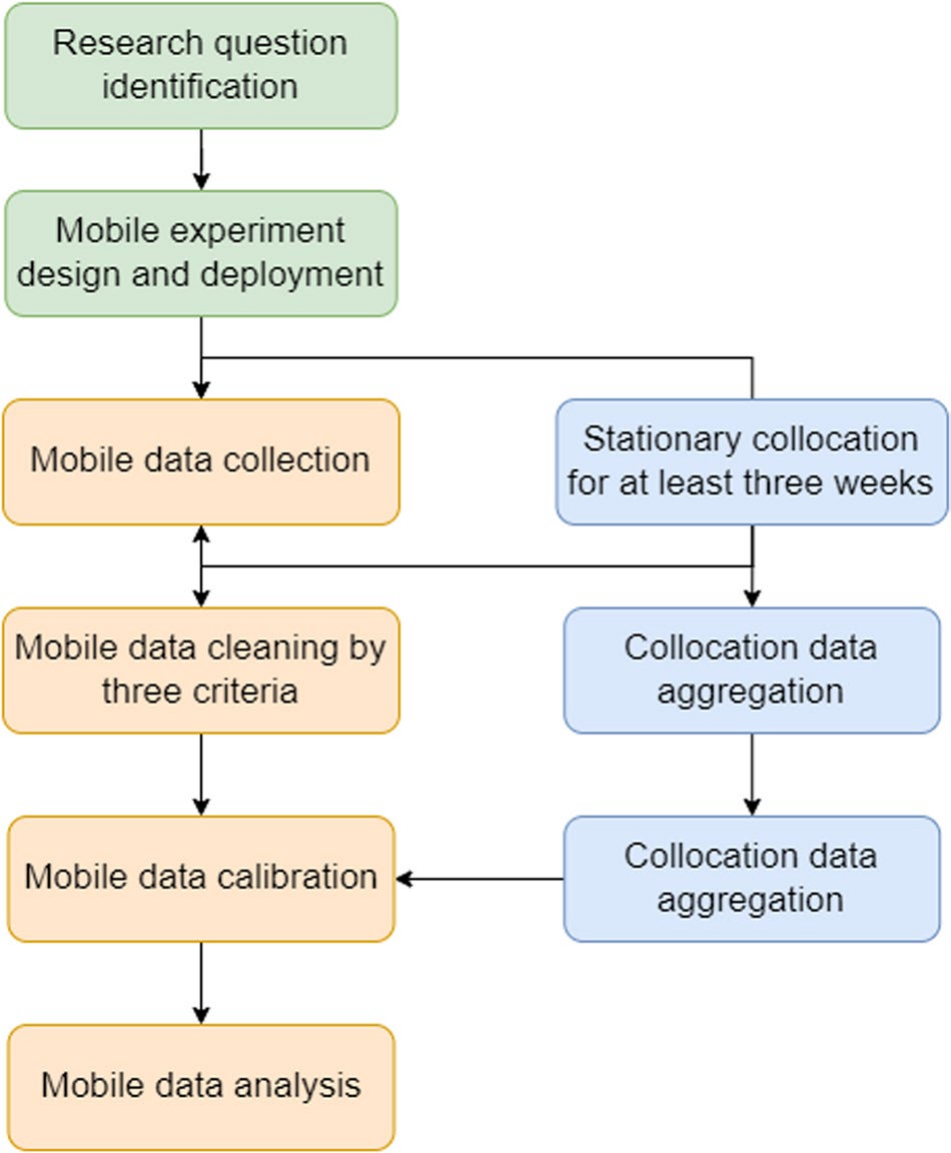
Data post-processing and validation protocols.

Low-cost sensors are prone to data quality and stability issues. For example, low-cost OPC cannot discern *particulate matter* from water droplets. Thus, it does not function well in high-humidity environments (85%)^19,20^. Therefore, it is necessary to perform sensor collocation and calibration to ensure accurate and robust measurements. Here we define collocation as the process of deploying low-cost sensors side-by-side with reference monitors and calibration as the adjustment of raw sensor readings using collocation data and mathematical models. Air quality datasets published in this study were cleaned, calibrated, and validated under a standardized framework referencing the US EPA air sensor performance testing protocols published in 2021^10,21,22^. US EPA is a federal agency that regulates and manages environmental protection matters. They also provide references, guidelines, and regulations considered “the gold standard” for air quality monitoring, primarily in the US and many other countries. A general flow chart of our post-processing and validation process is presented in Fig. 3.

Immediately before or after mobile deployments longer than three months, we perform stationary collocation for at least three weeks, as reference stations usually report measurements every hour, so that we can get around five hundred coincident data points for calibration model development. We suggest using collocation data with higher temporal resolution, ideally by minute, for modeling training, validating, and testing, given that CS readings are every five seconds. The models are statistical models developed using meteorological factors and CS readings as explanatory variables and reference monitor readings as the target variable. That is, to “calibrate” CS devices towards the reference. Empirically, we use mainly four meteorological factors from a central weather station for all CS units circulating in a city, including air temperature, humidity, air pressure, and dew point. For each CS unit, we developed a unit-specific calibration model to account for the inter-sensor variability of low-cost sensors, where each sensor responds to the same air pollutant concentration slightly different from the others. We adopted two types of tree-based calibration models, namely random forest and gradient boosting tree, which have faster training speed and better interpretability. Specifically, the second deployment in NYC employed the gradient boosting tree calibration model, as there is more collocation data (27,000 minute-level readings per CS unit compared to an average of 2,000 in other deployments). The other deployments were calibrated with random forest model, which provides better accuracy and better generalizability with less collocation data. Model training was realized in Python using scikit learn^23^ and LightGBM^24^ libraries and detailed hyperparameter tuning information can be found in our recent publication^10^.

Calibration models’ performance is evaluated with k-fold cross-validation^23^ and two statistical metrics, the r and the root mean squared error (RMSE), as shown in Eqs. 1, 2. r is the Pearson correlation coefficient between calibrated CS readings and reference readings. RMSE measures the absolute difference between these two readings. In the training and test datasets, the CS readings for air pollutants and meteorological factors, including temperature, humidity, dew point and air pressure, were used as explanatory factors, while the reference monitor readings were treated as the target factor. Other meteorological factors that are commonly found in local weather stations, including wind speed, wind direction, and feel temperature, were also tested in the models but removed due to statistical insignificance.

1 $$r=\frac{{\sum }_{i=1}^{n}({y}_{i}-\mathop{y}\limits^{-})({\widehat{y}}_{i}-\overline{\widehat{y}})}{\sqrt{{\sum }_{i=1}^{n}{({y}_{i}-\mathop{y}\limits^{-})}^{2}{\sum }_{i=1}^{n}{({\widehat{y}}_{i}-\overline{\widehat{y}})}^{2}}}$$

2 $$RMSE=\sqrt{\frac{{\sum }_{i=1}^{n}{\left({y}_{i}-\widehat{{y}_{i}}\right)}^{2}}{n}}$$

Where:

*y*_*i*_ and ${\widehat{y}}_{i}$ are observed and predicted target features in the test dataset;

$\mathop{y}\limits^{-}$ and $\overline{\widehat{y}}$ are mean values of observed and predicted target features in the test dataset;

*n* is the size of the test dataset.

The calibration models were then applied to cleaned mobile air quality data to produce the datasets we report in this paper. Our data cleaning process is straightforward, following the principle to preserve as many data points as possible. First, all sensors are functioning, yielding numerical results. CS is designed to give out “Not Applicable (NA)” signals when sensors operate with anomalies, such low battery or over-heating. Second, readings under high humidity (>90% or raining) are excluded, given that the low-cost particle counter we used is known to have skewed responses in this condition^19,25^. Lastly, we eliminate records with readings out of the reasonable ranges (<1 ug/m^3^ or >1000 ug/m^3^ for *PM*_*2.5*_, <200 mv or >900 mv for *NO*_*2*_ electro-signal). The reasonable ranges are determined by a priori knowledge of the ambient environment and the sensors themselves^26,27^. In total, about 15% of raw data are excluded in data cleaning.

*PM*_*2.5*_ calibration models’ performance for all cities is presented in Table 3. It is worth noticing that the calibration model performs poorly on the Beirut dataset, which is caused by Beirut’s limited air monitoring resources. Ideally, CS should be calibrated against reference-grade instruments before deployment, whose purchase and maintenance often require a good amount of financial input from governments and agencies. However, Beirut currently does not have enough resources to operate reference air quality monitoring station due to an economic collapse nor stand-alone reference-grade air instruments that we can access. Alternatively, we used a research-grade *PM* sensor for collocation and calibration, namely a Met One E-BAM Portable Environmental Beta-Attenuation Mass Monitor provided by our local collaborators. While the monitor is high-quality, it cannot meet the standards of reference monitors. It reveals that limited public investment for air quality monitoring and regulation can in turn lead to bigger air regulation and research gaps in less-developed regions. Our design of the CS platform and publication of our air monitoring data aim to narrow these gaps. Still, certain limitations exist in our approach.

**Table 3:** *PM*_*2.5*_ calibration model performance against reference or research-grade monitors.

Deployment	r	RMSE (µg/m^3^)
Pilot 1, New York City, US	0.96	—
Pilot 2, New York City, US	0.97	0.96
Boston, US	0.85	0.76
Beirut, Lebanon	0.68	5.38

## Data Records

**Fig. 4:**
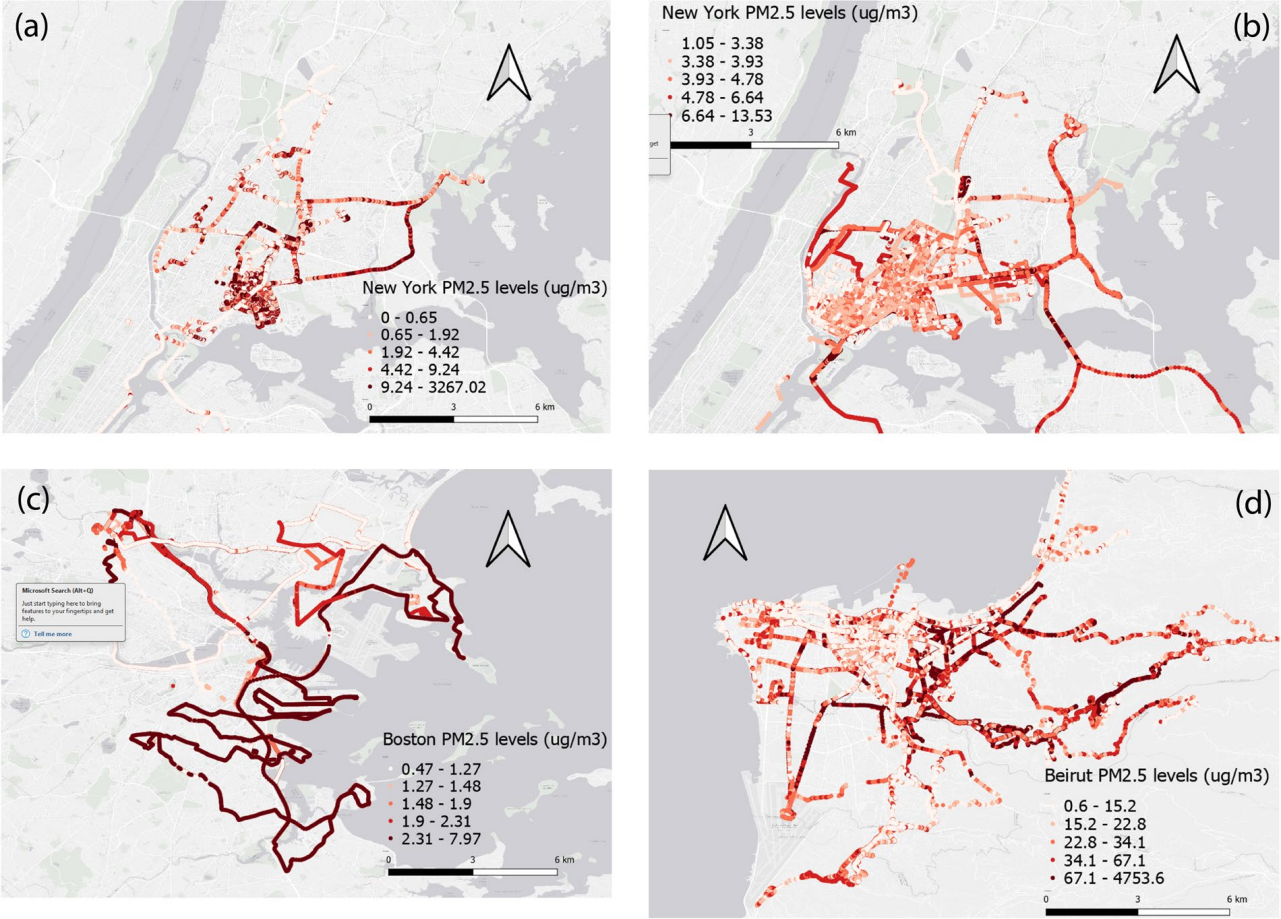
Spatial distributions of *PM*_*2.5*_ concentrations in (**a**) New York Pilot 1, (**b**) New York Pilot 2, (**c**) Boston, and (**d**) Beirut.

Data records from all devices for the same environmental indicator (e.g., *PM*_1_*, PM*_*2.5*_*, PM*_10_, and *NO*_2_) in each deployment are pooled in the same dataset. After data cleaning and calibration, there are 118,765 (NYC pilot 1), 515,917 (NYC pilot 2), 123,192 (Boston), and 56,628 (Beirut) 5s-interval data records. Each data file incorporates five categories of fields: unique sensor IDs, time stamp, GPS coordinates, weather, and calibrated concentrations, as demonstrated in Table 4. The number of fields for each deployment is determined by how many pollutants we have measured and calibrated. We map the spatial distributions of *PM*_2*.5*_ concentrations in the four deployments in Fig. 4. The datasets are available under Creative Commons Attribution license at Zenodo^28^.

**Table 4:** Data fields’ definitions and units. Note that not all fields are present in all datasets.

Field name	Definition	Unit
SensorID	Unique sensor IDs in each deployment	—
time	Time in Unix time	second
latitude, longitude	GPS location readings in WGS84 coordinates	degree
pm1, pm25, pm10	*Particulate matter* (*PM*) readings after calibration	µg/m^3^
no2	*Nitrogen dioxide* (*NO*_*2*_) readings after calibration	ppb
bin0-bin23	Twenty-four bins from the OPC containing particle number counts for different sizes from 0.35 to 40 µm. The cutting sizes are: 0.35, 0.46, 0.66, 1.0, 1.3, 1.7, 2.3, 3.0, 4.0, 5.2, 6.5, 8.0, 10.0, 12.0, 14.0, 16.0, 18.0, 20.0, 22.0, 25.0, 28.0, 31.0, 34.0, 37.0, 40.0	Number/second
temperature	Ambient temperature readings	Celsius
humidity	Ambient relative humidity	Percentage

## Usage Notes

Hyperlocal air quality data provide unique opportunities for data-driven environmental and climate decision-making. While covering a large area with high spatial resolution, mobile monitoring is subject to higher uncertainty than stationary monitoring, as the sensor only captures a snapshot of a certain location. Therefore, it is crucial to have repetitive measurements over the same location. It is suggested that at least four randomized measurements on different days should be aggregated to generate an average value of air pollution at a certain location in the same season^7^. In practice, a certain location can be defined as street segments or grid cells of different sizes. Given the different data collection duration and intensity across our deployments, the grid size we aggregated our observations with ranges from 100 by 100 m to 300 by 300 m. As a rule of thumb, at least 500 grids should cover at least one-third of a city’s land area with more than ten observations from at least four different days in the same season to generate a robust air quality surface. This ensures a good spatial representation of a city with representative levels of air pollution. Here we present a couple of potential applications of our air quality datasets, with a special focus on the New York City dataset and its application in air pollution mapping.

**Fig. 5:**
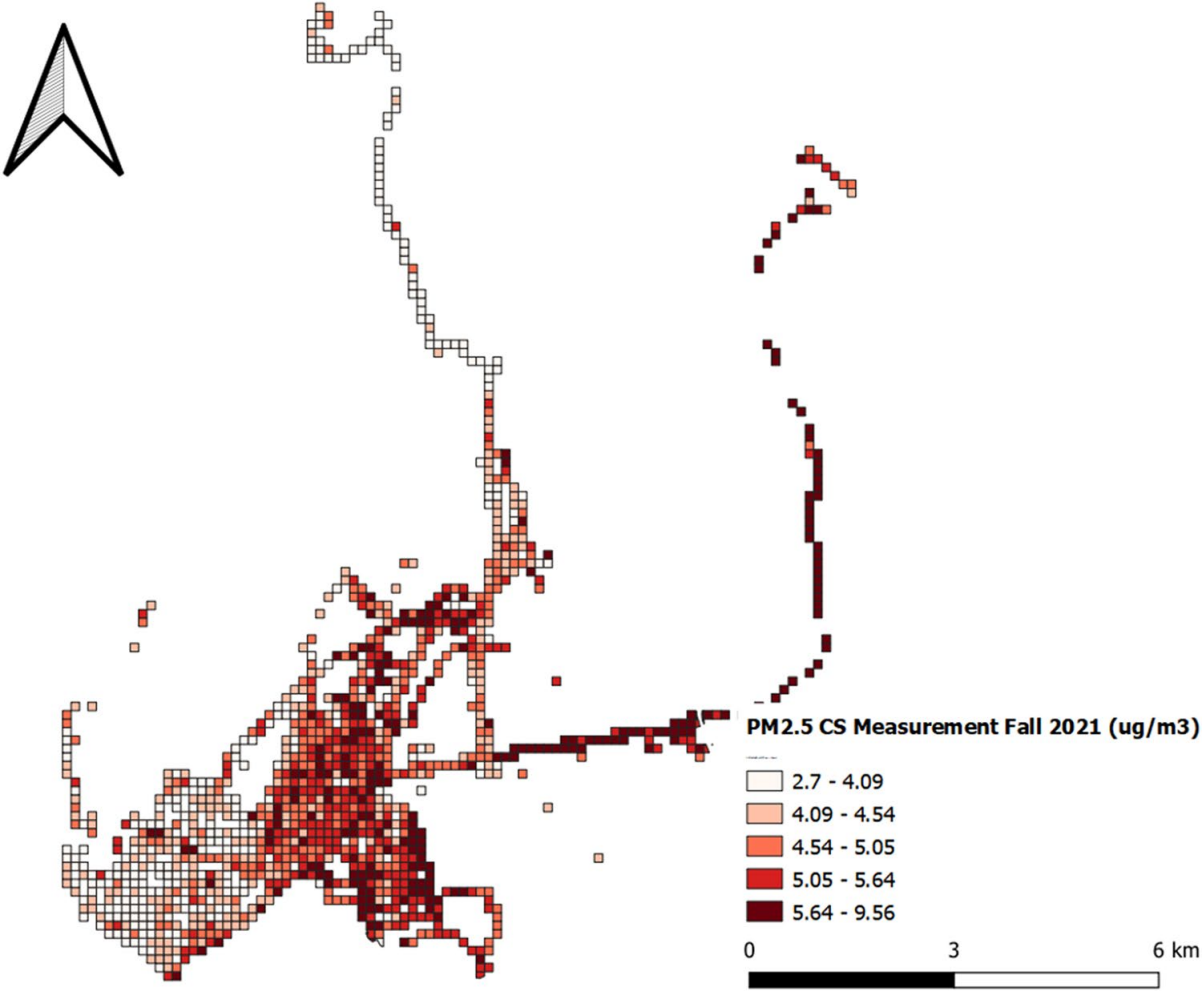
Measured *PM*_*2.5*_ concentrations in the Bronx after calibration and aggregation.

First, we look at the calibrated mobile measurement data in the Bronx, New York City. We aggregated the data in 100 by 100 m grid cells. All cells in Fig. 5 contain at least ten observations from 4 different days. This map is useful for identifying emission and air pollution hotspots, where we observe that highways and industrial areas in the bottom right corner suffer from significantly higher *PM*_2*.5*_ concentrations.

**Fig. 6:**
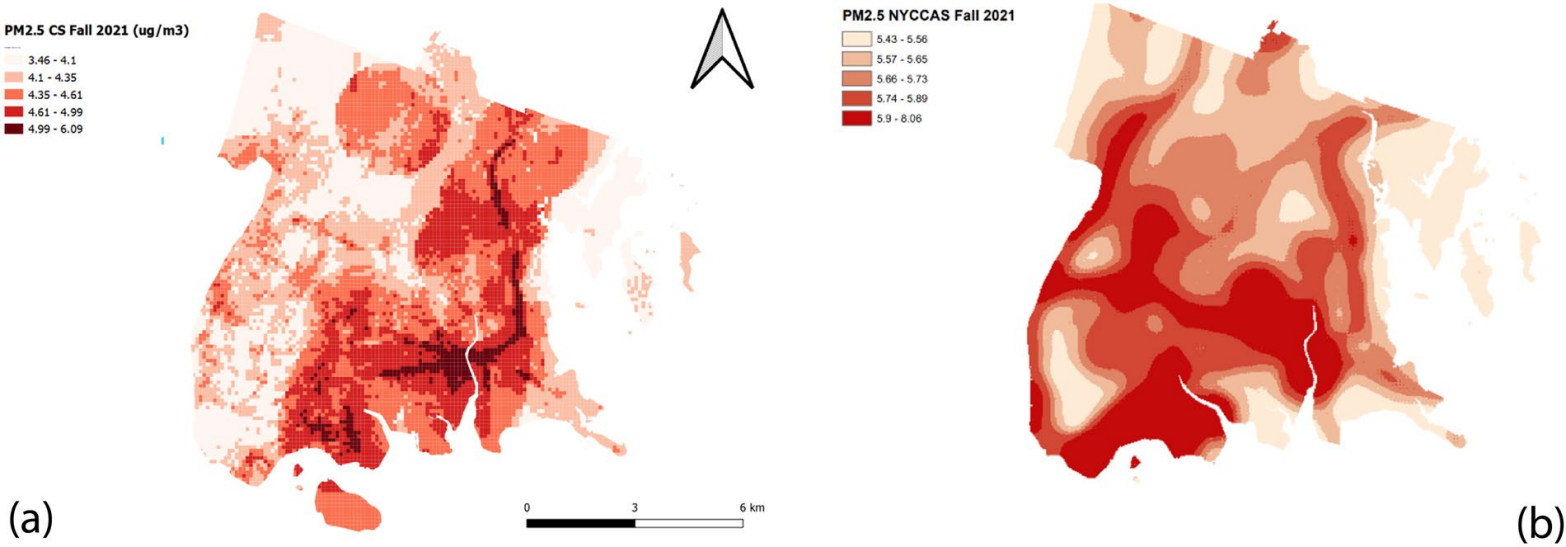
Land use regression surfaces of *PM*_*2.5*_ concentrations in the Bronx by (**a**) City Scanner data and (**b**) NYCCAS data.

Another common usage for mobile air quality data is land use regression (LUR), a spatial regression technique using the proximity of land use and emission indicators to explain and estimate air pollution levels in places without measurement. Using the measured *PM*_2*.5*_ concentrations in each grid cell, we create buffers of different sizes, ranging from 50 to 1000 m, and extract land use and emission indicators within each buffer size. The indicators are documented in Table 5, which align with the regulatory air pollution maps developed under the New York City Community Air Survey (NYCCAS) program and are retrieved from New York’s open data platform (https://nyccas.cityofnewyork.us/nyccas2021v9/sites/default/files/NYCCAS-appendix/Appendix1.pdf). Our prediction map presented in Fig. 6 illustrates a spatial pattern similar to the NYCCAS regulatory one in the same temporal scope, even though the absolute air pollution levels differ slightly. We further contrasted our *PM*_2*.5*_ predictions in 100 by 100 m grid cells with NYCCAS predictions cell by cell. We employed 2020 data for comparison, given the data unavailability for 2021 at a fine resolution. The Pearson correlation is 0.38 between the two datasets and the root mean square error is 2.02 µg/m^3^. Given that the 2020 NYCCAS predictions are also modeling results trained on observations, a relatively low correlation between the two models is anticipated. Still, the statistics demonstrate satisfactory performance of our prediction model and thus, the validity of our mobile sampling method and data quality assurance.

**Table 5:** Emission indicators used in air quality prediction maps.

Predictor category	Predictors examined (calculated in buffers of 50 to 1000 m)	Data source
Traffic indicators	Annual average daily traffic (AADT)	New York State (NYS) Department of Transportation (DOT) AADT, 2019
	Truck AADT	NYS DOT AADT, 2019
	Distance to nearest road by functional class	New York City (NYC) Open Data street centerline, 2022
	Road density by functional class	NYC Open Data street centerline, 2022
	Distance to nearest truck route	NYC Open Data truck route, 2021
	Truck route density	NYC Open Data truck route, 2021
	Distance to nearest bus route	NYC Open Data bus route, 2021
	Bus route density	NYC Open Data bus route, 2021
	Distance to nearest railroad	NYC Open Data railroad line, 2018
	Railroad density	NYC Open Data railroad line, 2018
Population metrics	Population density	US decennial census, 2020
Built space indicators	Density of built space (building floor area)	NYC Department of City Planning Primary Lan Use Tax Lot Output (PLUTO), 2022
	Density of residential units	PLUTO, 2022
	Total residential, industrial, commercial floor area	PLUTO, 2022
Land use predictors	Area of industry and manufacturing	PLUTO, 2022
	Area of open space & outdoor recreation	PLUTO, 2022
	Dominant land use type	PLUTO, 2022
Permitted emissions	Number of permitted combustion sources by fuel type (oil 2, 4, 6, natural gas)	NYC Department of Environmental Protection Clean Air Tracking System permit data, 2022
	Total BTU by fuel type (oil 2, 4, 6, natural gas)	NYC Mayor’s Office of Sustainability Local Law 84 Benchmarking data, 2017
Facilities	Number of transportation facilities (bus depots, terminals, ports, railyards, airports)	NYC Open Data City Planning Facilities Database (FacDB), 2022
	Distance to nearest transportation facilities	FacDB, 2022
	Number of waste transfer stations, waste processing sites, water treatment facilities	FacDB, 2022
	Distance to nearest waste transfer station waste transfer stations, waste processing sites, water treatment facilities	FacDB, 2022

Users are encouraged to develop more insights from our air quality datasets, calibration models, and use cases. This section presents potential future research directions here for future discussion and efforts. Firstly, it is useful for researchers and practitioners to look further into the causes of emission and air pollution hotspots, which might not align spatially, to reflect on their current emission reduction and air quality management strategies. Secondly, by overlaying spatial distributions of air quality and public health indicators, such as asthma rate, emergency room visits, and incidence of other respiratory and cardiovascular diseases, one can track down the adverse health impacts of air pollution from an epidemiological perspective. Lastly, it is interesting to investigate air pollution exposure and its disparity among communities through a static home location-based analysis or a dynamic mobility pattern-based analysis.

Here we acknowledge a few known shortcomings of the published datasets. Firstly, the low-cost OPC we use for *PM* measurement is known for its deteriorated performance in high-humidity environments, as it cannot differentiate *PM* from water droplets in the air. Therefore, our datasets do not include observations collected in >90% relative humidity. Secondly, the Boston and Beirut data sets were calibrated with research-grade sensors rather than reference-grade ones. The research-grade sensors used in Boston were calibrated at a reference station immediately before the mobile deployment. In Beirut, the research-grade sensors were the only available option for local calibration, given that no government-regulated reference stations existed. We do not consider this would lead to significant biases in the published datasets. Thirdly, our temperature and humidity data have not been calibrated against reference monitors as they are not the main focus of our deployments. In this case, their validity has not been adopted as a criterion for data cleaning, which aims to preserve the maximal number of valid observations for *particulate matter* and *NO*_*2*_. We highly advice the audience to only adopt them for educational or making sense purposes and to conduct a sanity check before any form of analysis. Lastly, given that CS is a low-cost environmental sensing platform, it is crucial to collocate and calibrate the platform before usage locally. This is especially important if a deployment is measuring *PM*, as the OPC counts particles in different-size bins and then estimates mass concentration with assumptions of the shape and density, which can vary significantly from place to place, from season to season.

## Data Availability

Other than air quality data stamped with time and location, we also provide a compilation of land use GIS layers that are used in our and NYCCAS’ LUR models for convenient reproduction of the results in our Github repository (https://github.com/MIT-Senseable-City-Lab/OSCS/tree/main). These GIS layers are published by NYC and New York State governments and processed by the authors for modeling, with 2021 as the base year. The audience is encouraged to explore the repository, regarding the details about how we design, build, calibrate, and make use of the CS platform. Python code is available for automatic land use feature extraction, LUR training, and performance evaluation.
